# Meaning Making Following Trauma

**DOI:** 10.3389/fpsyg.2022.844891

**Published:** 2022-03-23

**Authors:** Crystal L. Park

**Affiliations:** Department of Psychological Sciences, University of Connecticut, Storrs, CT, United States

**Keywords:** meaning, appraisal, cognitive processing, beliefs, goals

## Introduction

The meaning making model provides a useful framework for integrating myriad existing meaning-related theories and empirical findings. In this overview, I describe this model, which comprises both global and situational (event-specific) aspects. Global meaning encompasses foundational beliefs, values and goals, and a subjective sense of meaningfulness while situational meaning entails the appraisal of an experience. When an experience is perceived as discrepant with global meaning, individuals experience distress and engage in a variety of efforts to make meaning of that experience. Meaning making is usually aimed at changing the meaning of the situation but can also involve changing global meaning (e.g., adopting a new way of understanding the world or new goals; i.e., *meaning made*). Successful meaning making reduces discrepancies between global meaning and individuals’ assigned meaning of the specific experience and restores harmony within their global meaning vis-à-vis their current experience.

**Figure 1:**
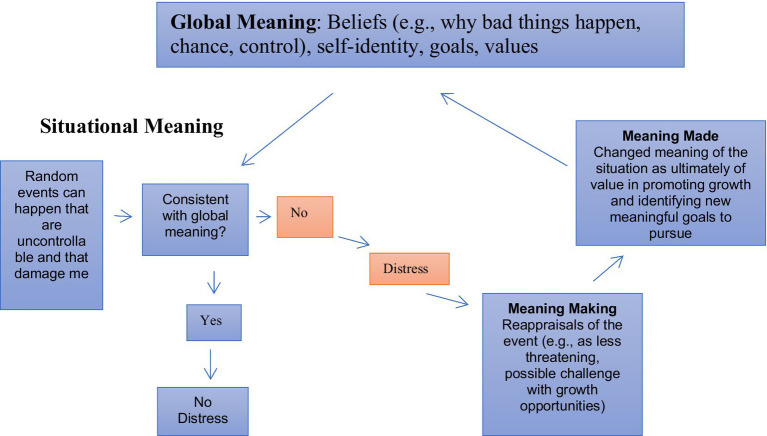
Meaning making model as applied to a negative situation.

The model of meaning making described here is based on a growing body of research regarding responses to adversity, such as serious illness, bereavement, sexual assault, incest, the COVID-19 pandemic, natural disasters, and terrorist attacks [Fitzke et al., 2021; see Park et al. (2017) and Park and Blake (2020), for reviews]. This model distinguishes two levels of meaning: global (people’s fundamental and overarching beliefs and their hierarchies of goals and values; Park, 2010) and situational (how global meaning, in conjunction with a given particular context, influences assigning meaning and responding to a particular situation; Park, 2017; Figure 1).

## Global Meaning

Global meaning refers to individuals’ foundational orienting systems (Trevino et al., 2019), consisting of individuals’ deeply held beliefs regarding reality, such as fairness, control, and identity (Park, 2017; Clifton et al., 2019; Pilkington et al., 2021) as well as their goals (states that one desires and pursues or that one already possesses and seeks to maintain, such as health, wealth, or family relationships; Lewis, 2020) In addition, global meaning includes a subjective sense of life as meaningful (e.g., purposeful, comprehensible; Park, 2010).

## Situational Meaning

In addition to global meaning systems, psychological adjustment is influenced by one’s circumstances and how those circumstances are understood (i.e., their situational meaning). People continuously monitor their experiences and assign meaning to (i.e., appraise) them. Encountering potentially difficult or stressful situations leads to determining the extent to which it is discrepant with one’s global meaning, and to the extent it is, coping with and making meaning of those experiences and adjusting to them.

### Appraisals of Events

People appraise, or assign a particular meaning, to their encounters, determining the extent to which they are threatening and controllable, attributing causes, and discerning their implications (Park, 2010). These appraised meanings, in turn, determine individuals’ reactions to those events. Highly traumatic events are commonly appraised as unpredictable, unfair, and uncontrollable and as having pervasive adverse implications for survivors and their futures (Brown et al., 2019). The meaning making model asserts that distress is not generated by the appraised meaning itself but rather by discrepancies between that appraised meaning and the individual’s global meaning system (Park, 2010; Park et al., 2016). For example, a study of pregnant women who experienced the Queensland Flood found that appraising the consequences of the flood on themselves and their families predicted later depression and anxiety symptoms, but appraising the consequences as positive buffered the long-term effects of peritraumatic distress on anxiety levels in these new mothers 2 years later (Paquin et al., 2021).

### Appraised Violations of Global Meaning

After people appraise or assign meaning to an event, they determine the degree to which it is consistent or discrepant with their global meaning. Perceived discrepancies (e.g., with their sense that the world is understandable and fair or that the event is not what they wanted to have happened) produce distress (Steger et al., 2015; Park et al., 2016). A scale to assess this global meaning violation was developed recently, the Global Meaning Violations Scale (GMVS; Park et al., 2016). A study of college students reporting on their most stressful event using the GMVS demonstrated that violations of global beliefs and violations of global goals were each independently related to distress. Similarly, a recent study of a national sample early in the COVID-19 pandemic showed that greater belief violations were associated with higher levels of anxiety and depression symptoms (Milman et al., 2020).

### Meaning Making

This violation-related distress is painful, motivating people to try to alleviate it. These efforts can involve meaning making, although people also engage in many other strategies to try to reduce their distress, including a variety of active and passive coping strategies (e.g., Park et al., 2021). Meaning making aims to restore disrupted global meaning through approach-oriented intrapsychic attempts to develop new and acceptable ways of understanding the situation that are more consistent with one’s global meaning or by changing one’s global meaning beliefs and goals. Following successful meaning making, people have a different view of the situation and have modified their beliefs and goals to regain consistency among them, an outcome termed *meaning made* (Park, 2017).

Severe trauma can disrupt a person’s global meaning (Janoff-Bulman, 1989). Making meaning typically involves cognitive processing of appraised and global meanings to change or reframe them and make them more consistent (Fitzke et al., 2021; Huang et al., 2021). People can change situational appraisals to better integrate them into their global meaning system (assimilation), such as coming to see the event as less damaging or, perhaps, even positive in its consequences (Paquin et al., 2021; Park and Boals, 2021). For appraisals of events that are highly discrepant with global meaning, meaning making may require changing one’s global meaning to accommodate the trauma.

Positive reappraisal, looking for ways to view the situation in a more positive light, or focusing on identifying positive attributes of an event and reminding oneself of those benefits, are very common meaning-making strategies (Park, 2010). A longitudinal study of a national sample of Americans (the Midlife in the United States Series Study) found that attempting to making meaning of a highly stressful or traumatic life event through positive reappraisal was associated with subsequent higher levels of positive mood and lower levels of negative mood years later (Fitzke et al., 2021). Other meaning making strategies include seeking more benign explanations for the situation and making downward comparisons with real or hypothetical others in relatively poorer straits (Gerber et al., 2018). Several studies of individuals dealing with the COVID-19 pandemic have highlighted some of the different strategies people use in efforts at making meaning of the pandemic, including through positive reinterpretation (Park et al., 2021), seeking out potential benefits or growth (Yang et al., 2021), and accepting the reality of the situation (Umucu and Lee, 2020).

### Meanings Made

Meaning making processes can be helpful by making new meanings, that is, changes in appraised or global meaning resulting from the cognitive processing involved in meaning making. Sometimes individuals change their understanding of the reason the event occurred, developing a more benign understanding; this new and more benign view is a key type of situational meaning made (e.g., Beierl et al., 2020). People may also perceive that they have changed in positive ways as a result of the trauma, such as improved relationships or enhanced coping skills (e.g., Park and Boals, 2021). Global beliefs and goals can change as well. For example, a study of Norwegian adults who survived the Southeast Asian tsunami in 2004 found those who reported their beliefs about the world changed in a positive way experienced fewer posttraumatic stress disorder (PTSD) symptoms and better quality of life (Nygaard and Heir, 2012). Survivors may change the goals they pursue as well, letting go of goals that are no longer realistic and doubling down on more attainable goals (Haase et al., 2021).

### Discrepancy Reduction Leads to Better Adjustment (Through Meanings Made)

People make meaning as a way to reduce discrepancies between situational and global meanings, and greater reductions in the size of discrepancies predicts better adjustment following trauma. For example, in a study of college students reporting on their most stressful or traumatic life event, reductions in global meaning violations over time was associated with concomitant reductions in PTSD symptoms (Park et al., 2016). Similarly, in a study of military veterans, reductions in Self-Blame through cognitive processing therapy, which relies strongly on meaning-making, were associated with reductions in PTSD symptoms (Holliday et al., 2018). On the other hand, continued inability to integrate one’s appraisal of a traumatic event into global meaning often leads to continued rumination, intrusive thoughts, and depression (Zakarian et al., 2019; Huang et al., 2021).

To date, findings from research conducted in many different trauma and stress contexts supports linkages among components of the meaning-making model. However, few studies have fully examined the set of linkages outlined in the meaning making model. For example, few studies have assessed violations of beliefs and goals nor whether meaning making efforts following trauma help make meaning by reducing discrepancies between appraised situational and global meaning. To adequately study these linkages would require longitudinal studies assessing initial situational appraisals, violations and distress, meaning making efforts, meanings made, changes in violations and subsequent adjustment. Instead, most of the work on meaning making is cross-sectional and retrospective (e.g., Huang et al., 2021), with only a few multiple-time point studies examining these issues (e.g., Fitzke et al., 2021). While this growing body of work suggests that discrepancy reductions mediate effects of meaning making and meanings made on adjustment, much remains to be learned about meaning making and its relations to managing and overcoming trauma.

## Conclusion

The meaning making model is a useful framework for integrating existing meaning-related theories and empirical findings (Park, 2010). To date, however, research has tested the model in piecemeal fashion, focusing on specific components or linkages, and findings are essentially supportive of the meaning making model. More inclusive longitudinal research focusing on relationships among various components as people engage in meaning making of and adjust to highly stressful situations will provide more rigorous tests of the model. Ultimately, the meaning making model will provide insight and clinical applications (Park et al., 2017) to promote better adjustment to highly stressful experiences.

## Author Contributions

CLP developed the ideas contained in this paper, conducted the literature review, and wrote and edited the manuscript.

## Conflict of Interest

The author declares that the research was conducted in the absence of any commercial or financial relationships that could be construed as a potential conflict of interest.

## Publisher’s Note

All claims expressed in this article are solely those of the authors and do not necessarily represent those of their affiliated organizations, or those of the publisher, the editors and the reviewers. Any product that may be evaluated in this article, or claim that may be made by its manufacturer, is not guaranteed or endorsed by the publisher.
